# Atrioventricular coupling and left atrial abnormality in type 2 diabetes mellitus with functional mitral regurgitation patients verified by cardiac magnetic resonance imaging

**DOI:** 10.1186/s12933-022-01536-2

**Published:** 2022-06-09

**Authors:** Yi Zhang, Xue-Ming Li, Meng-Ting Shen, Shan Huang, Yuan Li, Zhi-Gang Yang

**Affiliations:** 1 Department of Radiology, West China Hospital, Sichuan University, 37# Guo Xue Xiang, Chengdu, 610041 Sichuan China; 2 Department of Radiology, School of Medicine, Sichuan Cancer Hospital and Institute, Sichuan Cancer Center, University of Electronic Science and Technology of China, 55# Lan 4 RenMing Road (South), Chengdu, 610041 Sichuan China

**Keywords:** Type 2 diabetes mellitus, Functional mitral regurgitation, Atrioventricular coupling, Cardiac magnetic resonance

## Abstract

**Background:**

Functional mitral regurgitation (FMR) in type 2 diabetes mellitus (T2DM) patients induced by left ventricular (LV) enlargement and mitral valve abnormality may aggravated the impairment in left atrial (LA) compliance. Thus, this study aimed to depict how FMR and LV dysfunction affect LA compliance in T2DM patients with FMR.

**Materials and methods:**

A total of 148 patients with T2DM and 49 age- and sex-matched normal controls underwent cardiac magnetic resonance examination. LA longitudinal strain and LA and LV functional indices were compared among controls and different T2DM patients. The multivariate analysis was used to identify the independent indicators of LA longitudinal strain.

**Results:**

T2DM Patients without FMR had a lower total LA empty fraction (LAEF) compared with the controls (all P < 0.05). T2DM patients with mild and moderate FMR showed increased LA volume (LAV) and LV volume while decreased LAEF, LA strain, and LV ejection fraction (P < 0.05). T2DM patients with severe FMR showed markedly increased LAV and LV volume while decreased LAEF, LA strain, and LVEF (P < 0.05). In T2DM patients with FMR, reservoir strain (εs) was independently correlated with LV end-diastolic volume (LVEDV) (β = − 0.334) and regurgitation degree (β = − 0.256). The passive strain (εe) was independently correlated with regurgitation degree (β = − 0.297), whereas the active strain (εa) was independently correlated with LVESV (β = − 0.352) and glycated haemoglobin (β = − 0.279).

**Conclusion:**

FMR may aggravate LA and LV dysfunction in T2DM patients. Regurgitation degree was an independent determinant of the εs and the εe, LVEDV was an independent determinant of the εs, and LVESV was an independent determinant of the εa in T2DM patients with FMR.

**Supplementary Information:**

The online version contains supplementary material available at 10.1186/s12933-022-01536-2.

## Background

Functional mitral regurgitation (FMR) is reported as the most common heart valve disease in type 2 diabetes mellitus (T2DM) and accounts for 32% of this population [1]. FMR in T2DM patients induced by left ventricular (LV) enlargement, mitral annular dilation, papillary muscle displacement and mitral valve insufficiency is a common comorbidity in T2DM patients and is independently associated with an increased risk of mortality risk [2]. Epidemiologic studies show that the mild FMR leads to a 3.3-fold and the moderate to severe FMR leads to a 5.1-fold increase in all-cause mortality of T2DM patients [3, 4]. The exploration of the FMR contribution to myocardial abnormalities is important to the clinical management and risk evaluation in T2DM patients.

Most previous studies on myocardial abnormalites in T2DM patients with FMR have focused on the LV. However, the left atrium (LA) plays an important role in LV filling and regulating cardiac output regulation during the whole cardiac cycle [5]. T2DM with FMR may have blood flowing back into the LA from the LV during ventricular systole, thereby aggravating the impaired LA compliance, and conversely affecting the LV function [4, 6]. LA function has been shown independently correlated with the risk of hospitalisation for heart failure and mortality and may provide information in LV diastolic function assessment [7–9]. The exploration of FMR contribution to myocardial abnormalities and atrioventricular coupling is important for the clinical management and risk evaluation of T2DM patients.

Decreased LA strain was detected in T2DM although the LA volume was normal [10]. However, data on impaired LA strain and LV diastolic function coupling in the population of T2DM with FMR and the atrioventricular coupling remains limited. Moreover, LA function measures were proved as a sensitive early markers of dysfunctional LV diastolic and impaired LA strain impairment as measured by speckle tracking echocardiography as one of the first signs of diastolic dysfunction [10, 11].

Cardiac magnetic resonance (CMR) can provide an accurate morphological and functional LA and LV estimation in a ‘one-stop-shop’ scan [12, 13]. Growing evidence has been proven that CMR-derived strain can offer superior multiplanar imaging and real-time tracking of myocardial deformation regardless of limited acoustic window and operator dependence [14–16]. Therefore, this study aimed to depict how FMR aggravates the impaired LA compliance and LV function and to investigate the atrioventricular coupling in T2DM patients with FMR.

## Materials and methods

### Study population

This study included 204 patients who are clinically diagnosed with T2DM (based on the current American Diabetes Association guidelines: patients with fasting plasma glucose levels of ≥ 7.0 mmol/L, random plasma glucose levels of ≥ 11.1 mmol/L, or glycated haemoglobin (HbA1c) levels of ≥ 6.5%) and who underwent CMR scans in our hospital from December 2017 to December 2020 [17]. A total of 56 patients were excluded from the study because of ischaemic heart disease, rheumatic heart disease, congenital heart disease, primary cardiomyopathy and other heart valve diseases, CMR contraindications, poor image quality and incomplete scan Finally, a total of 148 T2DM patients with an average age of 58.2 ± 10.8 years and a body mass index (BMI) of 24.7 ± 3.7 kg/m^2^ were included. Among them, 54 (36.5%) cases had FMR (17 mild cases, 18 moderate cases and 19 severe cases) and 94 (63.5%) cases had no FMR (Additional file 1). Moreover, 49 age- and sex-matched normal individuals were enrolled in the control group [34 males and 15 females; mean age: 55.5 ± 6.8 years; BMI: 22.5 (20.4, 24.7) kg/m^2^].

Clinical data, including gender, age, height, weight, blood pressure, resting heart rate, fasting plasma glucose (FPG), HbA1c, months with diabetes, total cholesterol, triglycerides, high-density lipoprotein, low-density lipoprotein and antidiabetic drugs (biguanides, sulfonylureas, α-glucosidase inhibitors, glucagon-like peptide-1/dipeptidyl peptidase-4 inhibitors, sodium-glucose cotransporter 2 inhibitors and insulin), were collected from the digital medical records. The baseline information of the control group was collected before the CMR scans. The BMI was calculated as weight (kg) divided by the square of height (m) [18]. Blood pressure and resting heart rate was recorded as an average of three measurements in the right arm in a sitting position after a 10-min resting period.

### CMR examination

We used a 3.0 T whole-body scanner (Skyra; Siemens Medical Solutions, Erlangen, Germany) with a 32-channel body phased-array coil to conduct a CMR scan in the supine position. A series of 8–12 continuous short-axis views of LV and a four- or two-chamber long-axis views of the left heart was obtained using steady-state free precession (slice thickness: 8.0 mm; field of view: 360 × 300 mm^2^; matrix size: 256 × 166 pixels; flip angle: 40°; repetition time: 2.81 ms; and echo time: 1.22 ms).

### Image post-processing

#### Volumetric and functional analysis

Two experienced radiologists performed the image post-processing on commercial software (cvi42; Circle Cardiovascular Imaging, Inc., Calgary, AB, Canada). Firstly, The endocardial contour of the LA, left and right ventricle was manually delineated in the end-diastolic and end-systolic images of the LV. Additionally, the long and short diameters of the LA were measured. LA volumetric analysis was performed according to the biplane area-length method. The system automatically calculated the maximum LA volume (LAVmax), LA volume prior to atrial contraction (LAVpac) and minimum LA volume (LAVmin). Then, LA function indices were calculated, including total LA emptying fraction (LAEF), passive LAEF and active LAEF [19]. The LV parameters, including LV end-diastolic volume (LVEDV), LV end-systolic volume (LVESV), LV stroke volume (LVSV), LV ejection fraction (LVEF), LV mass and right ventricluar stroke volume (RVSV), were also automatically computed according to the standardized image interpretation and post-processing [20].

#### LA strain analysis

Tissue tracking technology was used to track each myocardial voxel on the horizontal 4-chamber long-axis and vertical 2-chamber long-axis cine slices. Then the software automatically analysed the global longitudinal strain indices of the LA, including reservoir strain (εs), passive strain (εe), active strain (εa), peak positive strain rate (SRs), peak early negative strain rate (SRe) and peak late negative strain rate (SRa) [19].

#### FMR diagnosis and classification in T2DM patients

The regurgitation signal could be identified on the short axis, four-chamber long axis and two-chamber long axis views on cine sequence as a high-speed black retrograde signal from the LV to the LA through the mitral valve during ventricular systole [21]. The regurgitation fraction (RF) was calculated by using LVSV and RVSV according to the following the formula: RF = (LVSV − RVSV)/LVSV [22]. T2DM patients with FMR were further divided into mild (RF of < 30%), moderate (RF of 30%–50%) and severe (RF of ≥ 50%) regurgitation according to the calculated results [14, 23].

### Reproducibility of LA strain

The intra-observer variability in the LA strain indices was assessed by an experienced investigator by comparing the measurements from 60 randomly selected cases analysed by the same observer after one month. The inter-observer variability was evaluated by comparing the measurements from the same population by another independent double-blinded experienced observer.

### Statistical analyses

All continuous variables are showed as the mean ± standard deviation. Comparisons between two groups were analysed using Student’s t-test or the Mann–Whitney U test as appropriate. Comparisons between more than two groups were made with one-way analysis of variance. The frequency (percentage) was used to represent the categorical variables, and the Chi-square test was used to compare the constituent ratio between different groups. Spearman’s test was used to analyse the correlation between LA strain and LV function indices. LV variables with P < 0.05 and no collinearity in univariate analysis along with clinical characters (FPG, HbA1c, months with diabetes, regurgitation degree) were included in the backward multiple linear regression model adjusted by age, BMI, and resting heart rate. Inter-observer and intra-observer variability of LA strain were assessed by the intraclass correlation coefficient (ICC). All analyses were performed by a two-tailed test, and P < 0.05 was considered statistically significant.

## Results

### Baseline characteristics of the study cohort

**Fig. 1:**
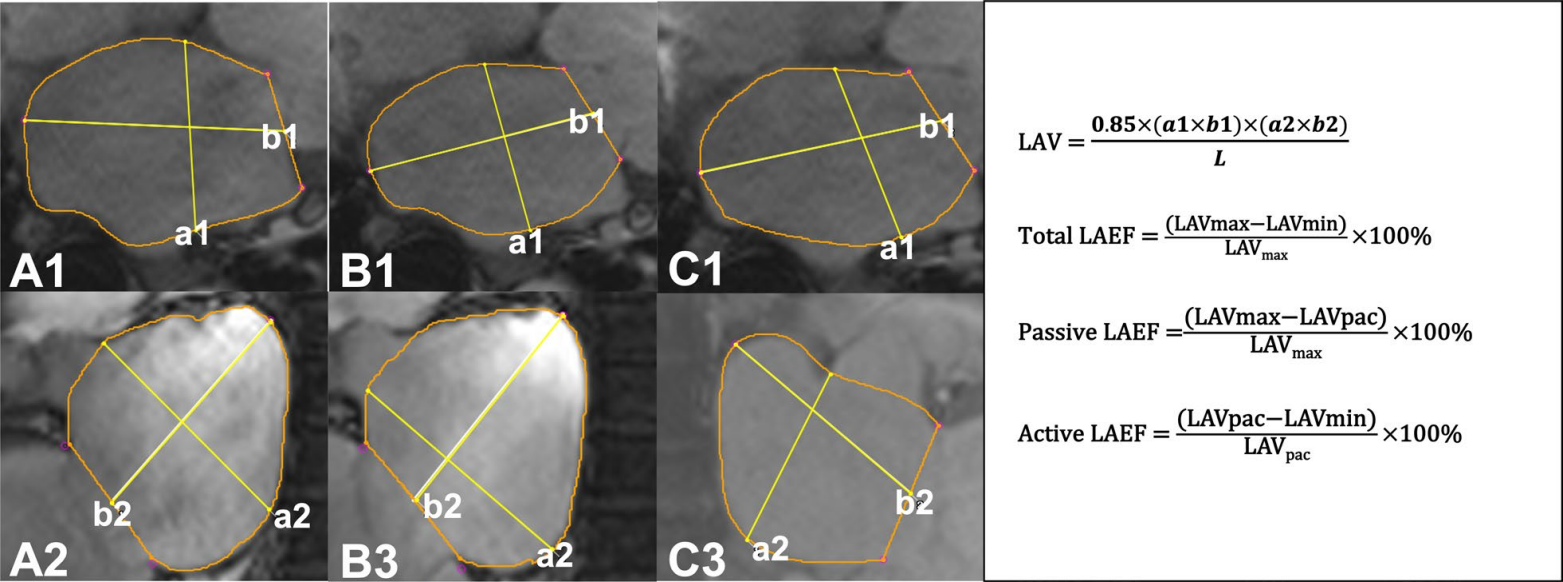
Calculation of left atrial volume and function. The left atrial endocardial contour (orange curve) was delineated and the long and short diameters (yellow line) of the LA were measured at the end of left ventricular systole (A1,2), before left atrial systole (B1,2) and end of left ventricular diastole (C1,2) of the four-chamber long axis (A1, B1, C1) and two-chamber long axis (A2, B2, C2) images of CMR slices. And the left atrial volume (LAV) and left atrial emptying fraction (LAEF) according to the formula. a: the short diameter of the left atrium; b: the long diameter of the left atrium; L: the shorter length of the long axis of the left atrium in two-chamber or four-chamber cardiac planes

Table 1 shows the baseline characteristics, fasting plasma glucose (FPG), glycated haemoglobin (HbA1c), months with diabetes, blood lipid levels, regurgitation degrees, and medication. T2DM patients with FMR were older [60.2 ± 11.9 years vs. 55.2 ± 6.8 years] and had significantly higher weight [66.7 ± 9.7 kg vs. 60.4 ± 10.7 kg] and BMI [25.4 ± 4.9 kg/m^2^ vs. 23.2 ± 3.7 kg/m^2^] compared with those in the normal control (P < 0.05). T2DM patients without FMR had higher weight [65.9 ± 10.7 kg vs. 60.4 ± 10.7) kg], BMI [24.3 ± 2.9 kg/m^2^ vs. 23.2 ± 3.7 kg/m^2^] and systolic blood pressure [128.7 ± 19.6 mmHg vs. 119.4 ± 5.2 mmHg] than the normal ones (P < 0.05). patients with FMR patients suffered through longer months with diabetes than patients without FMR patients [12.8 ± 5.5 months vs. 8.3 ± 7.0 months, P < 0.05] (Fig. 1).

**Table 1:** Clinical characteristics of the study population

	Normal (n = 49)	T2DM	
		Without FMR (n = 94)	With FMR (n = 54)
Gender, male (%)	34 (69.4)	65 (69.1)	34 (62.9)
Age, years	55.2 ± 6.8	57.1 ± 9.9	60.2 ± 11.9a
Height, cm	161.0 ± 6.4	164.5 ± 8.5	162.8 ± 10.5
Weight, kg	60.4 ± 10.7	65.9 ± 10.7a	66.7 ± 9.7a
BMI, kg/m^2^	23.2 ± 3.7	24.3 ± 2.9a	25.4 ± 4.9a
SBP, mmHg	119.4 ± 5.2	128.7 ± 19.6a	125.7 ± 25.3
DBP, mmHg	79.9 ± 7.6	79.9 ± 12.6	76.9 ± 18.6
Rest heart rate, bmp	74.1 ± 10.0	77.0 ± 12.3	77.8 ± 14.6
FPG, mmol/L	–	9.8 ± 4.9	9.3 ± 4.9
HbA1c, %	–	7.9 ± 1.9	8.2 ± 1.5
Months with diabetes, months	–	8.3 ± 7.0	12.8 ± 5.5b
TC, mmol/L	–	4.6 ± 1.9	4.2 ± 1.7
TG, mmol/L	–	2.1 ± 2.3	2.0 ± 1.4
HDL, mmol/L	–	1.2 ± 0.5	1.0 ± 0.4
LDL, mmol/L	–	2.5 ± 1.3	2.4 ± 1.2
T2DM with mild regurgitation, n (%)	–	–	17(31.5)
T2DM with moderate regurgitation, n (%)	–	–	18 (33.3)
T2DM with severe regurgitation, n (%)	–	–	19 (35.2)
Medication, n			
Biguanides	–	49	25
Sulfonylureas	–	6	8
α-Glucosidase inhibitor	–	21	14
GLP-1 receptor and DPP-4 inhibitors	–	9	8
SGLT-2 inhibitors	–	12	5
Insulin	–	33	19

a, *P* < 0.05, T2DM vs. Normal; T2DM, type 2 diabetes mellitus; FMR, functional mitral regurgitation; BMI, body mass index; SBP, systolic blood pressure; DBP, diastolic blood pressure; FPG, fasting plasma glucose; HbA1c, glycated hemoglobin; TC, total cholesterol; TG, triglycerides; HDL, high-density lipoprotein; LDL, low-density lipoprotein

**Fig. 2:**
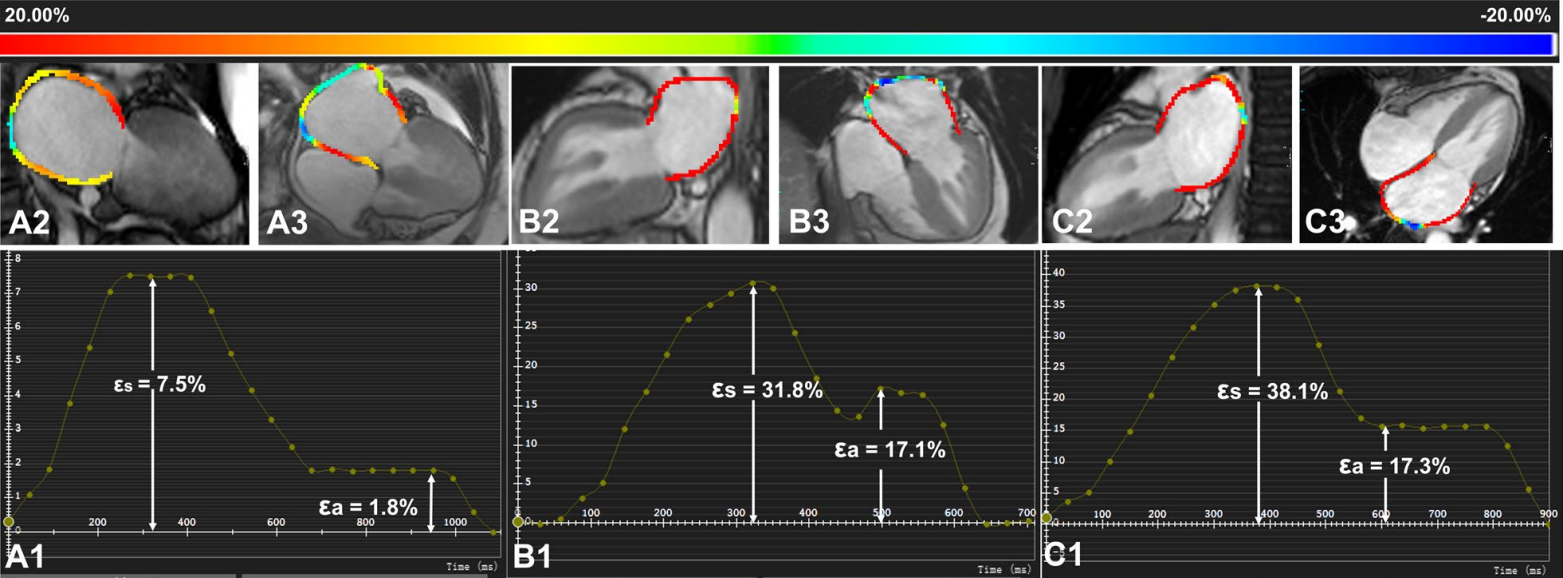
CMR cine pseudo-colour images and strain-time curves of LA strain in normal individuals and T2DM patients with and without FMR. A1-3: T2DM with FMR, female, 64 years old, εs = 7.5%, εa = 1.8%; B1-3: patient with T2DM without FMR, male, 56 years old, εs = 31.8%, εa = 17.1%; C1-3: normal individual, female, 51 years old, εs = 38.1%, εa = 17.3%. CMR, cardiac magnetic resonance; T2DM: type 2 diabetes mellitus; FMR: functional mitral regurgitation; εs: left atrial reservoir strain; εa: left atrial active strain

Table 2 showed the comparison among normal individuals and T2DM patients with different FMR stages. Figure 2 showed CMR cine pseudo-colour images and CMR-derived strain curves of LA strain in normal individuals and T2DM patients with and without FMR.

**Table 2:** Comparison of left atrial and ventricular CMR characteristics among study groups

	Normal (n = 49)	T2DM			
		Without FMR (n = 94)	With mild FMR (n = 17)	With moderate FMR (n = 18)	With severe FMR (n = 19)
LA parameters					
LAVmax, mL	57.8 ± 16.2	63.9 ± 26.5	98.8 ± 33.7ab	97.2 ± 41.7ab	149.9 ± 90.3abcd
LAVpac, mL	39.7 ± 13.0	46.4 ± 23.5	80.9 ± 32.1ab	79.2 ± 39.0ab	128.0 ± 95.0abcd
LAVmin, mL	23.1 ± 7.8	29.4 ± 19.3	59.9 ± 29.2ab	62.2 ± 35.6ab	106.9 ± 95.1abcd
Total LAEF, %	60.0 ± 8.4	52.2 ± 14.5a	41.6 ± 13.3a	39.8 ± 19.0ab	32.1 ± 19.5abc
Passive LAEF, %	30.4 ± 19.4	27.7 ± 15.3	19.6 ± 6.4a	20.3 ± 10.0a	13.2 ± 9.4ab
Active LAEF, %	38.9 ± 22.3	39.0 ± 14.2	27.5 ± 15.5a	22.4 ± 16.9a	28.4 ± 21.6ab
LA strain, %					
ε_s_	48.4 ± 16.9	39.9 ± 18.4a	24.5 ± 10.3ab	22.0 ± 15.2ab	14.7 ± 11.2abc
ε_e_	30.1 ± 14.7	19.3 ± 9.3a	9.2 ± 5.1ab	8.9 ± 5.4ab	6.4 ± 5.5abc
ε_a_	18.3 ± 7.1	20.7 ± 12.3	15.3 ± 9.0a	13.1 ± 11.2ab	8.3 ± 6.7abc
LA SR (1/s)					
SRs	0.4 ± 2.8	1.2 ± 2.0	0.8 ± 1.1	1.2 ± 0.7	0.9 ± 0.6
SRe	− 2.4 ± 0.9	− 1.9 ± 1.4a	− 1.3 ± 0.9a	− 1.0 ± 0.8ab	− 0.6 ± 0.6abc
SRa	− 0.6 ± 2.6	− 1.3 ± 2.2	− 1.0 ± 1.6	− 1.4 ± 1.1	− 1.0 ± 1.2
LV parameters					
LVEDV	122.2 ± 27.1	132.7 ± 42.8	182.2 ± 64.1a	200.6 ± 78.2ab	214.6 ± 85.9ab
LVESV	45.4 ± 12.6	54.1 ± 30.7	104.6 ± 52.0ab	125.6 ± 84.4ab	140.4 ± 83.1abc
LVSV	76.9 ± 18.7	78.6 ± 23.0	77.7 ± 17.0	80.5 ± 31.2	74.2 ± 23.1
LVEF	62.6 ± 5.9	60.6 ± 10.1	45.6 ± 11.6ab	45.3 ± 19.3ab	40.2 ± 19.5ab
LVM	73.1 ± 19.2	96.2 ± 32.5a	121.6 ± 338.9ab	116.5 ± 45.0ab	108.3 ± 37.8a

a, *P* < 0.05, compared to normal; b, *P* < 0.05, compared to T2DM without FMR; c, *P* < 0.05, compared to T2DM with mild FMR; T2DM, type 2 diabetes mellitus; FMR, functional mitral regurgitation; LA, left atrium; LV, left ventricle; LVEDV, left ventricle end-diastolic volume; LVESV, left ventricle end-systolic volume; LVSV, left ventricle stroke volume; LVEF, left ventricle ejection fraction; LVM, left ventricle mass; LAVmax, maximum left atrial volume; LAVpac, left atrial volume prior to atrial contraction; LAVmin, minimum LA volume; LAEF, left atrium emptying fraction; εs, left atrial reservoir strain; εe, left atrial passive strain; εa, left atrial active strain; SRs, peak positive strain rate; SRe, peak early negative strain rate; SRa, peak late negative strain rate

### Comparison of CMR indices between T2DM patients without FMR and normal individuals

T2DM without FMR had lower total LAEF [52.2 ± 14.5% vs. 60.0 ± 8.4%], εs [39.9 ± 18.4% vs. 48.4 ± 16.9], lower εe [19.3 ± 9.3% vs. 30.1 ± 14.7%] and SRe [− 1.9 ± 1.4 1/s vs. − 2.4 ± 0.9 1/s]. For LV indices, only LVM was significantly increased than normal individuals [91.6 (71.8, 115.0) g vs. 73.1 ± 19.2 g, P < 0.05]. The rest indices in T2DM patients without FMR were similar as that in normal individuals (all P > 0.05).

### Myocardial abnormalities of LA and LV in T2DM patients with mild FMR

T2DM patients with mild FMR had increased LAVmax (98.8 ± 33.7 mL vs. 63.9 ± 26.5 mL, 57.8 ± 16.2 mL), LAVpac [80.9 ± 32.1 mL vs. 46.4 ± 23.5 mL, 39.7 ± 13.0 mL] and LAVmin [59.9 ± 29.2 mL vs. 29.4 ± 19.3 mL, 23.1 ± 7.8 mL] while decreased εs [24.5 ± 10.3 vs. 39.9 ± 18.4%, 48.4 ± 16.9) %] and εe [9.2 ± 5.1% vs. 19.3 ± 9.3%, 30.1 ± 14.7%] compared both with patients without FMR and normal individuals (all P < 0.05). Besides, passive LAEF [19.6 ± 6.4% vs. 30.4 ± 19.4%], active LAEF [27.5 ± 15.5% vs. 38.9 ± 22.3%] and εa [15.3 ± 9.0% vs. 18.3 ± 7.1%] began to decline in patients with mild FMR compared to normal individuals (all P < 0.05). For LV indices, patients with mild FMR had obviously increased LVEDV (182.2 ± 64.1 mL vs. 122.2 ± 27.1 mL), increased LVESV [104.6 ± 52.0 ml vs. 45.4 ± 12.6 mL], increased LVM [121.6 ± 338.9 g vs. 73.1 ± 19.2 g] and decreased LVEF (45.6 ± 11.6% vs. 62.6 ± 5.9%) compared to normal individuals and T2DM patients without FMR (all P < 0.05).

### Myocardial abnormalities of LA and LV in T2DM patients with moderate FMR

T2DM patients with moderate FMR had progressively decreased total LAEF [39.8 ± 19.0% vs. 52.2 ± 14.5%, 60.0 ± 8.4%], εa [13.1 ± 11.2%, 20.7 ± 12.3%, 18.3 ± 7.1%], and SRe [− 1.0 ± 0.8 1/s vs. − 1.9 ± 1.4 1/s, − 2.4 ± 0.9 1/s] compared to both T2DM patients without FMR and normal individuals (all P < 0.05). For LV indices, patients with moderate FMR had progressively increased LVEDV [200.6 ± 78.2 mL vs. 132.7 ± 42.8 mL, 122.2 ± 27.1 mL] and decreased LVEF [45.6 ± 11.6% vs. 60.6 ± 10.1%, 62.6 ± 5.9%] compared to both patients without FMR and normal individuals (all P < 0.05).

### Myocardial abnormalities of LA and LV in T2DM patients with severe FMR

For LA indices, T2DM patients with severe FMR had marked increase in LAVmax, LAVpac and LAVmin compared with normal individuals, patients without FMR, and T2DM with mild/moderate FMR (all P < 0.05). Moreover, patients with severe FMR had marked decrease in total LAEF (32.1 ± 19.5% vs. 41.6 ± 13.3%, 52.2 ± 14.5%, 60.0 ± 8.4%), εs [14.7 ± 11.2% vs. 24.5 ± 10.3%, 39.9 ± 18.4%, 48.4 ± 16.9%], εe [6.4 ± 5.5% vs. 9.2 ± 5.1%, 19.3 ± 9.3%, 30.1 ± 14.7%], εa [8.3 ± 6.7% vs. 15.3 ± 9.0%, 20.7 ± 12.3%, 18.3 ± 7.1%] and SRe [-0.6 ± 0.6 1/s vs. − 1.3 ± 0.9 1/s, − 1.9 ± 1.4 1/s, − 2.4 ± 0.9 1/s) than patients with mild FMR, patients without FMR and normal individuals respectively (all P < 0.05). Additionally, the passive LAEF [13.2 ± 9.4% vs. 27.7 ± 15.3%, 30.4 ± 19.4%] and active LAEF [28.4 ± 21.6% vs. 39.0 ± 14.2%, 38.9 ± 22.3%] were progressively decreased compared with T2DM patients without FMR and normal subjects (all P < 0.05).

For LV indices, T2DM patients with severe FMR had markedly increased LVEDV [214.6 ± 85.9 mL vs. 132.7 ± 42.8 mL, 122.2 ± 27.1 mL] and LVESV [140.4 ± 83.1 mL vs. 54.1 ± 30.7 mL, 45.4 ± 12.6 mL] while decreased LVEF [40.2 ± 19.5% vs. 60.6 ± 10.1%, 62.6 ± 5.9%] compared to both T2DM patients without FMR and normal individuals (all P < 0.05).

### Influencing factors of LA strain in T2DM patients with FMR

**Fig. 3:**
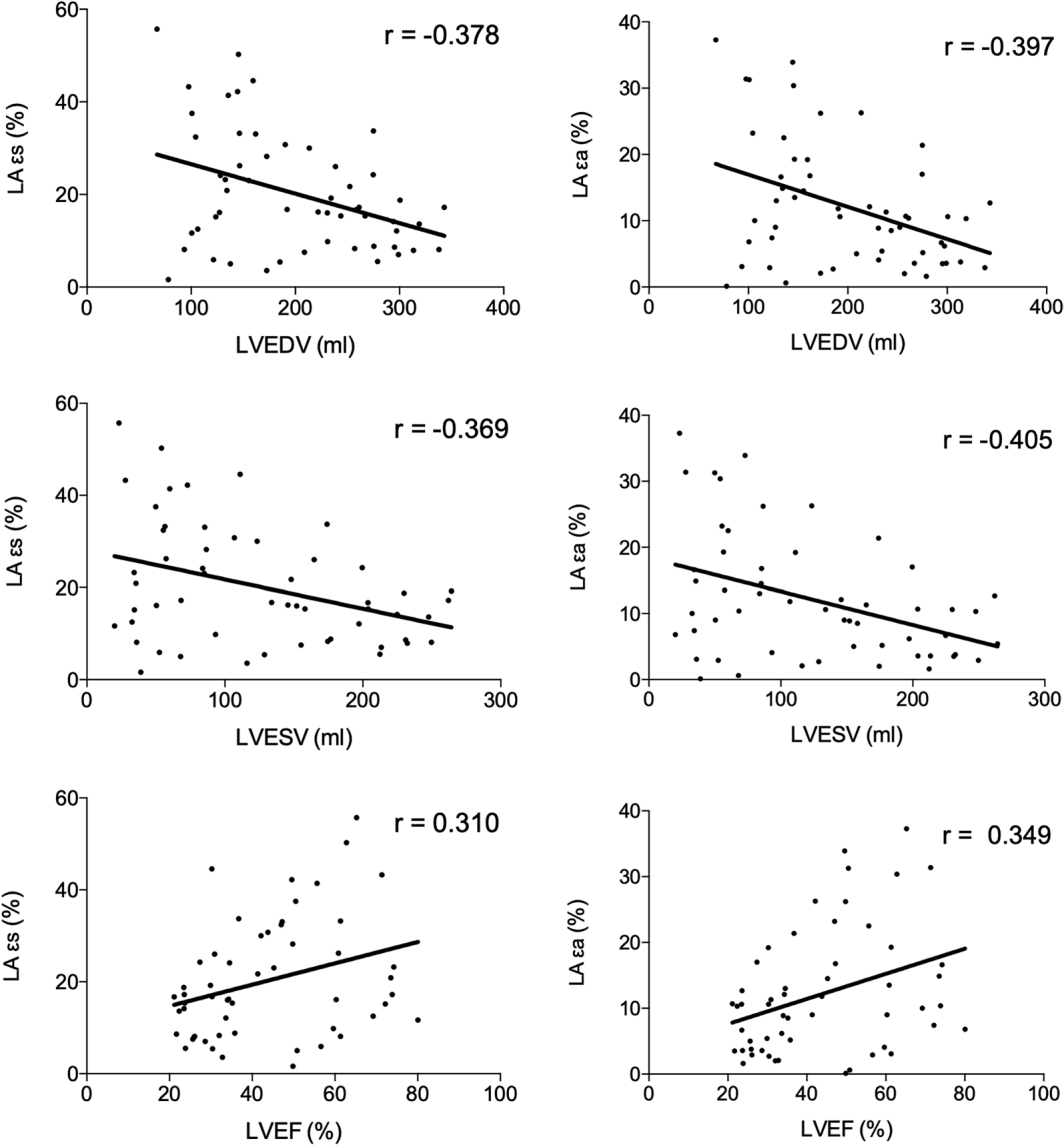
Correlations between the εs or the εa and LV function indices. LA, left atrium; εs, left atrial reservoir strain; εa, left atrial active strain; LVEDV, left ventricle end-diastolic volume; LVESV, left ventricle end-systolic volume; LVEF, left ventricle ejection fraction; r, correlation coefficient

Firstly, the εs was negatively correlated with LVEDV (R = -0.378, P < 0.05) and LVESV (R = -0.369, P < 0.05) and positively correlated with LVEF (R = 0.310, P < 0.05) in T2DM patients with FMR (P < 0.001). No correlation was found between εs and LVSV and LVM (P = 0.677, 0.361, respectively). Moreover, there was no correlation between εe and LV indices (P = 0.124, LVEDV; 0.202, LVESV; 0.768, LVSV; 0.337, LVEF; 0.361, LVM; respectively). Additionally, The εa was negatively correlated with LVEDV (R = − 0.397, P < 0.05) and LVESV (R = -0.405, P < 0.05), and positively correlated with LVEF (R = 0.349, P < 0.05)) in T2DM patients with FMR (all P < 0.05). No correlation was found between the εa and LVSV and LVM (P = 0.687, 0.459, respectively). Figure 3 showed scatter plots that illustrates the correlations between the εs or the εa and LV function indices.

The multivariate analysis showed that the εs was independently correlated with the LVEDV [β = − 0.334; 95% confidence intervals (CI) = − 0.099 to − 0.013] and regurgitation degree [β = − 0.256; 95% CI = − 7.993 to − 0.027]. The εe was independently correlated with the regurgitation degree (β = − 0.297; 95% CI = − 3.872 to − 0.007). Finally, the εa was independently correlated with the LVESV (β = − 0.352; 95% CI = − 0.074 to − 0.014) and the HbA1c (β = − 0.279; 95% CI = − 3.259 to − 0.255) (Table 3).

**Table 3:** Multivariate analysis between LA strain and regurgitation degree or LV indices in T2DM with FMR patients

	ε_s_ (Adjusted R^2^ = 0.207)			ε_e_ (Adjusted R^2^ = 0.189)			ε_a_ (Adjusted R^2^ = 0.242)		
	β	P	95% CI for B	β	P	95% CI for B	β	P	95% CI for B
FPG	0.123	0.335	–	0.058	0.686	–	0.124	0.329	–
HbA1c	− 0.213	0.088	–	− 0.069	0.634	–	− 0.279	0.023	− 3.259, − 0.255
Months with diabetes	− 0.003	0.983	–	− 0.154	0.275	–	0.080	0.526	–
Regurgitation degree	− 0.256	0.049	− 7.993, − 0.027	− 0.297	0.049	− 3.872, − 0.007	− 0.234	0.058	–
LVEDV	− 0.334	0.011	− 0.099, − 0.013	–	–	–	− 0.069	0.838	–
LVESV	− 0.061	0.861	–	–	–	–	− 0.352	0.005	− 0.074, − 0.014
LVEF	0.063	0.739	–	–	–	–	0.021	0.939	–

The regression model was adjusted by age, BMI, and heart rate. P < 0.05 in univariate analysis and entered into the multivariate model. T2DM, type 2 diabetes mellitus; FMR, functional mitral regurgitation; LA, left atrium; LV, left ventricle; LVEDV, left ventricle end-diastolic volume; LVESV, left ventricle end-systolic volume; LVEF, left ventricle ejection fraction; εs, left atrial reservoir strain; εe, left atrial passive strain; εa, left atrial active strain; CI, confidence intervals. There was no correlation between LVSV / LVM with LA strain, thus they did not enter the multivariate model

### Reproducibility of LA PS

ICC analysis showed that CMR tissue tracking technique was effective in measuring LA εs (within observer: ICC = 0.959, 95% CI = 0.948–0.959; between observer: ICC = 0.886, 95% CI = 0.816–0.934), εe (within observer: ICC = 0.948, 95% CI = 0.911–0.971; between observer: ICC = 0.858, 95% CI = 0.774–0.918), εa (within observer: ICC = 0.950, 95% CI = 0.916–0.972; between observer: ICC = 0.865, 95% CI = 0.783–0.921), SRs (within observer: ICC = 0.914, 95% CI = 0.853–0.952; between observer: ICC = 0.779, 95% CI = 0.660–0.868), SRe (within observer: ICC = 0.894, 95% CI = 0.820–0.941; between observer: ICC = 0.739, 95% CI = 0.604–0.842) and SRa (within observer: ICC = 0.903, 95% CI = 0.834–0.946; between observer: ICC = 0.755, 95% CI = 0.627–0.853).

## Discussion

This study verified that FMR may aggravate myocardial abnormalities in T2DM patients, and further characterized the atrioventricular coupling in T2DM patients with FMR. The following were demonstrated: (1) T2DM patients without FMR had normal LV function and impaired LA reservoir and conduit function compared with normal individuals. (2) FMR may aggravate LA and LV impairment and further deteriorate active LA function. (3) The LA enlargement and dysfunction were synchronous with LV dysfunction. (4) The regurgitation degree was an independent determinant of the εs and the εe. The LVEDV was an independent determinant of the εs. The LVESV was an independent determinant of the εa.

T2DM is susceptible to coexist with multi-system dysfunction, with cardiac dysfunction being one of the primary comorbidity. Sustained hyperglycaemia causes myocardial extracellular matrix deposition, increases myocardial cell oxygen consumption of myocardial cells, myocardial hypertrophy and myocardial interstitial fibrosis, leading to the myocardial overall deterioration [24]. Previous studies have been reported the LA morphological and functional abnormalities in T2DM patients [25, 26]. This study proved that T2DM patients with normal morphology also had significantly decreased total LAEF, εs, εe, and SRe, which suggests that the most obvious LA dysfunction induced by T2DM mainly occurs in the reservoir and conduit phase. LV stiffness increases in the early stage and LV compliance decreases when T2DM is not accompanied by FMR, resulting in declined blood flow from the LA to the LV in the early and middle diastole, leading to LA enlargement, decreased reservoir and conduit strain and maintained active strain, to compensate for the LV filling volume in the late diastole [27, 28].

Secondly, we found that FMR may aggravate LA and LV dysfunction. We found enlarged LA and LV in T2DM patients with mild FMR, meanwhile, the εa, active LAEF and LVEF began to decline. Moreover, LA compliance is synchronized with LV dysfunction and progressively decreased with increased regurgitation degree. The blood flowed back into the LA and rapidly raised the venous pressure and volume when T2DM is combined with FMR. The myometrium length of the LA myocardium exceeds the optimal length, the LA contractile decreases and the function of the LA in each phase reduces [28]. It was quite different from the strain characteristics of the LA in patients with primary mitral regurgitation. Borg et al. [29] reported that the strain of LA in the reservoir, conduit and active phase increased to in different degrees in patients with chronic primary mitral regurgitation. This further confirmed that the way of LA myocardial impairment in T2DM patients with FMR is progressive and without compensation.

Moreover, multivariate analysis revealed that the regurgitation degree was an independent determinant of the εs and the εe, the LVEDV was an independent determinant of the εs, and the LVESV and the HbA1c were both independent determinants of the εa. The atrioventricular coupling has been reported by Steele et al. in adolescents and young adults who are obese and with T2DM. They demonstrated an independent association between LA strain with LV diastolic function [10]. Additionally, Sun et al. also proved that the major cause of LA remodelling is elevated LV filling pressure [30]. Thus, focusing on the FMR contribution to myocardial abnormalities and the atrioventricular coupling may facilitate the clinical management and risk evaluation of T2DM patients.

This study has several limitations. Firstly, this is a single-centre study, but the sample size is adequate to support the conclusion. Secondly, this study did not conduct a long-term follow-up, and no case of atrial fibrillation was observed in our patients. Therefore, the prognostic effect of LA strain on atrial fibrillation in T2DM patients will be studied in the future. Finally, this study did not obtain pathological specimens to confirm the relationship between the LA mechanical changes and LA myocardial fibrosis; thus, related animal experiments will be conducted in the future.

In conclusion, FMR may aggravate impaired LA compliance and LV function in T2DM patients with regurgitation degree progression. The regurgitation degree was an independent determinant of the εs and the εe, and the LVEDV was an independent determinant of the εs, and the LVESV was an independent determinant of the εa.

## Supplementary Information

**Additional file 1.**The flow chart of the participant selection process of this study.

## Data Availability

The datasets used and analyzed during the current study are available from the corresponding author on reasonable request.

## Abbreviations

FMR: Functional mitral regurgitation
T2DM: Type 2 diabetes mellitus
LV: Left ventricular
LA: Left atrial
CMR: Cardiac magnetic resonance
FPG: Fasting plasma glucose
HbA1c: Glycated haemoglobin
HDL: High density lipoprotein
LDL: Low density lipoprotein
LVEDV: Left ventricle end-diastolic volume
LVESV: Left ventricle end-systolic volume
LVSV: Left ventricle stroke volume
LVEF: Left ventricle ejection fraction
LVM: Left ventricle mass
RVSV: Right ventricle stroke volume
LAVmax: Maximum left atrial volume
LAVpac: Left atrial volume prior to atrial contraction
LAVmin: Minimum LA volume
LAEF: Left atrium emptying fraction
RF: Regurgitation fraction
εs: Left atrial reservoir strain
εe: Left atrial passive strain
εa: Left atrial active strain
SRs: Peak positive strain rate
SRe: Peak early negative strain rate
SRa: Peak late negative strain rate
ICC: Intraclass correlation coefficient
CI: Confidence interval
