# Supplementary figures and images for: Atrioventricular coupling and left atrial abnormality in type 2 diabetes mellitus with functional mitral regurgitation patients verified by cardiac magnetic resonance imaging
Source: Cardiovasc Diabetol. 2022 Jun 9;21:100. doi: 10.1186/s12933-022-01536-2 (PMC9185866; doi:10.1186/s12933-022-01536-2)

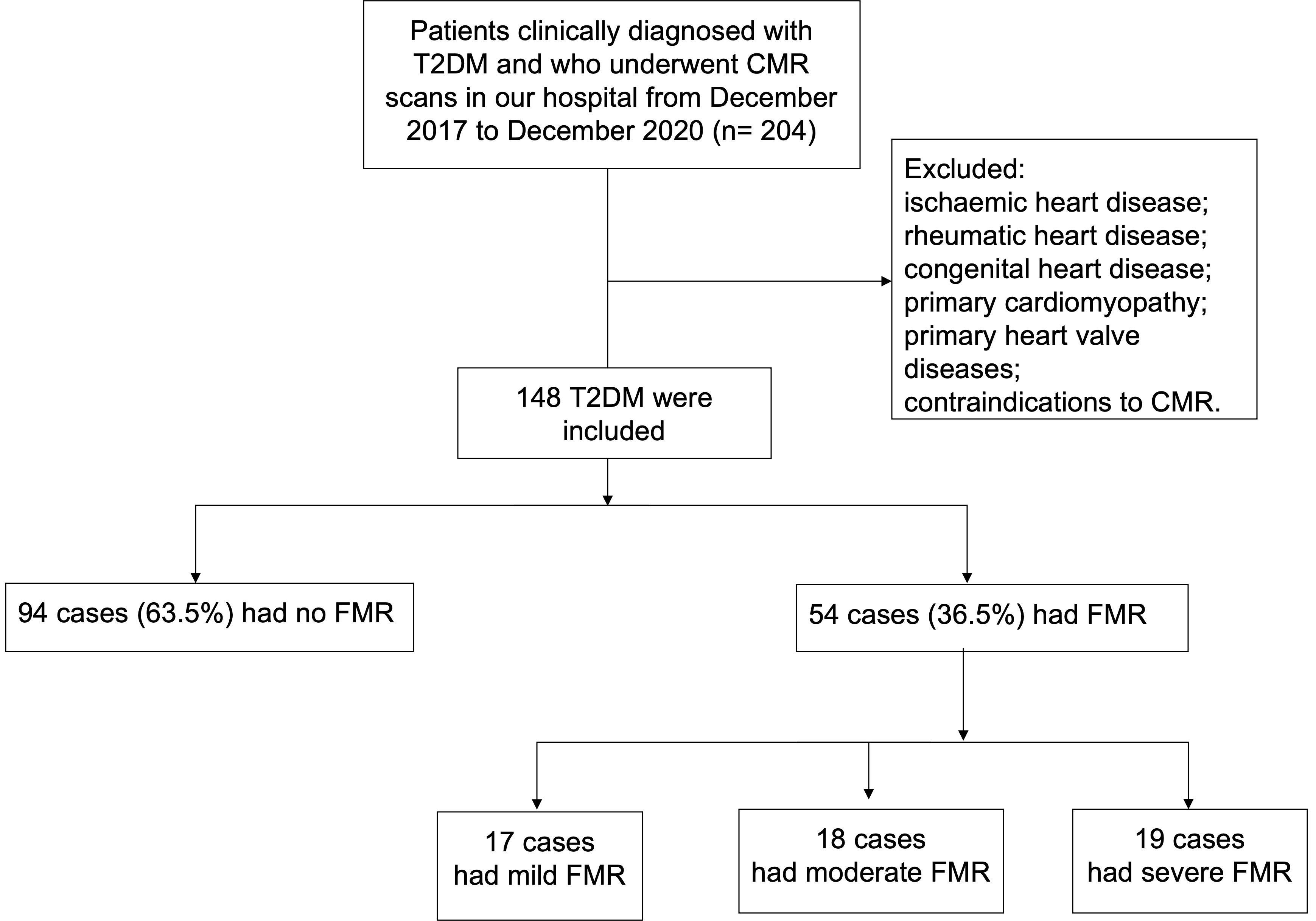

Supplement: Supplementary file 1 — Additional file 1.The flow chart of the participant selection process of this study. [file 12933_2022_1536_MOESM1_ESM.tif]
